# Direct observation of nodeless superconductivity and phonon modes in electron-doped copper oxide Sr_1−x_Nd_x_CuO_2_

**DOI:** 10.1093/nsr/nwab225

**Published:** 2021-12-15

**Authors:** Jia-Qi Fan, Xue-Qing Yu, Fang-Jun Cheng, Heng Wang, Ruifeng Wang, Xiaobing Ma, Xiao-Peng Hu, Ding Zhang, Xu-Cun Ma, Qi-Kun Xue, Can-Li Song

**Affiliations:** State Key Laboratory of Low-Dimensional Quantum Physics, Department of Physics, Tsinghua University, Beijing 100084, China; State Key Laboratory of Low-Dimensional Quantum Physics, Department of Physics, Tsinghua University, Beijing 100084, China; State Key Laboratory of Low-Dimensional Quantum Physics, Department of Physics, Tsinghua University, Beijing 100084, China; State Key Laboratory of Low-Dimensional Quantum Physics, Department of Physics, Tsinghua University, Beijing 100084, China; State Key Laboratory of Low-Dimensional Quantum Physics, Department of Physics, Tsinghua University, Beijing 100084, China; State Key Laboratory of Low-Dimensional Quantum Physics, Department of Physics, Tsinghua University, Beijing 100084, China; State Key Laboratory of Low-Dimensional Quantum Physics, Department of Physics, Tsinghua University, Beijing 100084, China; State Key Laboratory of Low-Dimensional Quantum Physics, Department of Physics, Tsinghua University, Beijing 100084, China; Frontier Science Center for Quantum Information, Beijing 100084, China; Beijing Academy of Quantum Information Sciences, Beijing 100193, China; RIKEN Center for Emergent Matter Science (CEMS), Wako, Saitama 351-0198, Japan; State Key Laboratory of Low-Dimensional Quantum Physics, Department of Physics, Tsinghua University, Beijing 100084, China; Frontier Science Center for Quantum Information, Beijing 100084, China; State Key Laboratory of Low-Dimensional Quantum Physics, Department of Physics, Tsinghua University, Beijing 100084, China; Frontier Science Center for Quantum Information, Beijing 100084, China; Beijing Academy of Quantum Information Sciences, Beijing 100193, China; Southern University of Science and Technology, Shenzhen 518055, China; State Key Laboratory of Low-Dimensional Quantum Physics, Department of Physics, Tsinghua University, Beijing 100084, China; Frontier Science Center for Quantum Information, Beijing 100084, China

**Keywords:** nodeless superconductivity, phonon modes, cuprate superconductors, CuO_2_ plane

## Abstract

The microscopic understanding of high-temperature superconductivity in cuprates has been hindered by the apparent complexity of crystal structures in these materials. We used scanning tunneling microscopy and spectroscopy to study the electron-doped copper oxide compound Sr_1−_*_x_*Nd*_x_*CuO_2_, which has only bare cations separating the CuO_2_ planes and thus the simplest infinite-layer structure of all cuprate superconductors. Tunneling conductance spectra of the major CuO_2_ planes in the superconducting state revealed direct evidence for a nodeless pairing gap, regardless of variation of its magnitude with the local doping of trivalent neodymium. Furthermore, three distinct bosonic modes are observed as multiple peak-dip-hump features outside the superconducting gaps and their respective energies depend little on the spatially varying gaps. As well as the bosonic modes, with energies identical to those of the external, bending and stretching phonons of copper oxides, our findings reveal the origin of the bosonic modes in lattice vibrations rather than spin excitations.

## Teaser

Tunneling conductance spectra of the essential CuO_2_ planes in the structurally simplest infinite-layer cuprate superconductors Sr_1-x_Nd_x_CuO_2_ revealed direct evidences for nodeless superconductivity and three lattice vibrational modes.

## INTRODUCTION

Despite more than three decades of intensive research, it remains a mystery how high-temperature (*T*_c_) superconductivity works in a family of ceramic materials known as cuprates [1,2]. In pursuit of its microscopic mechanism, two fundamental prerequisites are needed to identify the superconducting energy gap (Δ) function and bosonic glue (e.g. lattice vibrations and spin excitations) to pair electrons of the copper oxide (CuO_2_) planes. In theory [3], the bosonic excitation (mode) often exhibits itself via a strong coupling to the paired electrons, in the low-lying quasiparticle states at energy *E* = Δ + Ω (Ω is the boson energy). This shows great promise for simultaneously measuring the Δ and Ω by tunneling spectroscopy, which has unequivocally established the phonon-mediated *s*-wave pairing state in conventional superconductors [4]. For cuprate superconductors, however, no consensus exists on both the superconducting gap symmetry and the pairing glue [2,5–12]. One explanation could be that previous tunneling data from surface-sensitive scanning tunneling microscopy (STM) have been mostly measured on various charge reservoir layers [8,10–12], where a nodal gap behavior probably associated with charge density wave often occurs [2,13]. Here we report a high-resolution STM study of the electron-doped cuprate compound Sr_1−_*_x_*Nd*_x_*CuO_2_ (SNCO, *x* ∼ 0.100) and directly reveal nodeless superconductivity and three distinct bosonic modes on the CuO_2_ planes. Our analysis of the bosonic mode energies, which depend little on the spatially varying Δ, supports a lattice vibrational origin of the modes consistent with external, bending and stretching phonons of the copper oxides.

Infinite-layer SrCuO_2_ is, structurally, one of the simplest cuprate parent compounds that comprises the essential CuO_2_ planes separated only by strontium atoms. Partial substitution of divalent strontium (Sr^2+^) by trivalent neodymium (Nd^3+^) ions leads to electron doping and superconductivity with a record electron-doped cuprate transition temperature *T*_c_ of 40 K [14]. Most importantly, the single-crystalline SNCO epitaxial films with well-controlled doping level *x*, grown on SrTiO_3_(001) substrates with an oxide molecular beam epitaxy (MBE), exhibit a rare surface termination of the essential CuO_2_ planes [15–17]. Tunneling spectra of the electron-doped infinite-layer cuprates, which have so far been largely unexplored compared to their hole-doped counterparts [8], pose considerable challenges and opportunities. The challenge is to clarify whether the electron-doped cuprates and direct measurements of the major CuO_2_ planes are fundamentally different from or analogous to their hole-doped counterparts and those of the charge reservoir planes, respectively, whereas the critical opportunity is that addressing these issues might help greatly in finding the culprit of high-*T*_c_ superconductivity in copper oxide superconductors.

## RESULTS

### Temperature-dependent resistivity in Sr_1−x_Nd_x_CuO_2_

**Figure 1.**
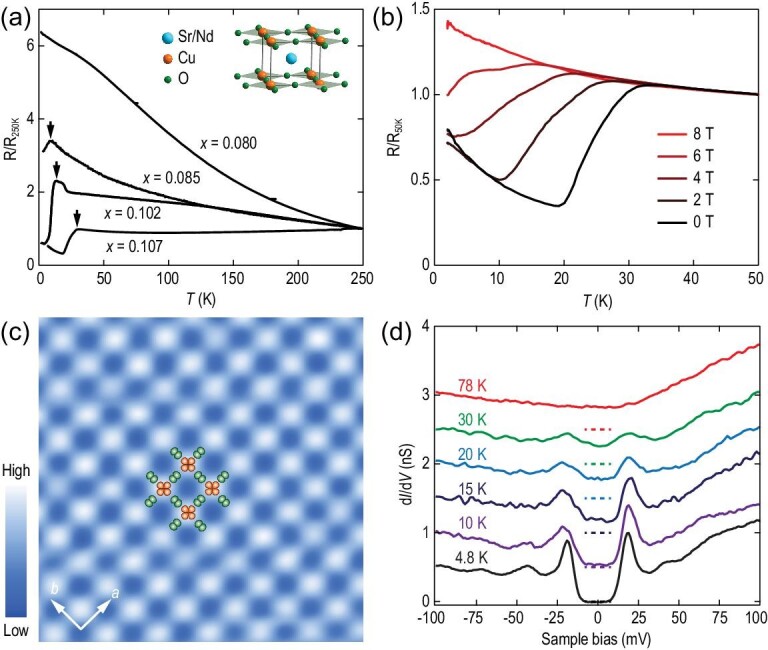
Characterizations of SNCO epitaxial films. (a) Temperature dependence of electrical resistivity, normalized to the value of 250 K, in electron-doped Sr_1−_*_x_*Nd*_x_*CuO_2_ (0.080 ≤ *x* ≤ 0.107) cuprate films, with a nominal thickness of ∼13 nm. Arrows denote the onset temperatures of superconductivity. Inset shows the schematic crystal structure of SNCO. (b) Electrical resistivity versus temperature of Sr_0.893_Nd_0.107_CuO_2_ measured under different magnetic fields, normalized to the value of 50 K for clarification. (c) Atom-resolution STM topography (3.6 nm × 3.6 nm, *V* = −1.5 V, *I* = 20 pA) of *x* ∼ 0.100 SNCO film. The bright spots denote the Cu atoms at the top layer. A single CuO_2_ plaquette with Cu 3d (orange) and O 2p (green) orbitals is shown. (d) Temperature dependence of differential conductance d*I*/d*V* spectra on the superconducting CuO_2_ plane. Setpoint: *V* = −200 mV and *I* = 100 pA.

Figure 1a plots the temperature dependence of the electrical resistivity of SNCO with varying Nd doping concentration *x*. An insulator–superconductor transition triggered by Nd^3+^ dopants becomes evident at *x* > 0.080. The superconducting phase is further unambiguously confirmed by applying an external magnetic field to the *x* = 0.107 SNCO sample in Fig. 1b. As anticipated, the electrical resistivity at low temperatures is elevated with increasing field until a complete suppression of superconductivity at 8 T. It is worth noting that the resistivity does not drop down to zero below *T*_c_. Instead, it exhibits an upturn behavior, which is later revealed by site-resolved tunneling spectroscopy to arise from nanoscale electronic phase separation between the superconductivity and under-doped Mott insulating state. Anyhow, the observed *T*_c_ onset (marked by the black arrows in Fig. 1a) up to 30 K turns out to be higher than those previously reported in epitaxial SNCO films on SrTiO_3_ substrates [18].

### Spectroscopic evidence of nodeless superconductivity and phonon modes

As demonstrated before [15–17], the heteroepitaxy of SNCO films on SrTiO_3_ proceeds in a typical layer-by-layer mode. Figure 1c shows a constant-current STM topographic image that displays an atomically flat and defect-free copper oxide surface in one SNCO sample of *x* ∼ 0.100. The adjacent Cu atoms are spaced ∼0.39 nm apart, which agrees with the previous reports [14–17]. In Fig. 1d, we show the energy-resolved tunneling conductance (d*I*/d*V*) spectra, being proportional to the quasiparticle density of states (DOS), directly on the CuO_2_ plane. At 4.8 K, the spectral weight is completely removed over a finite energy range around the Fermi level (*E*_F_), and instead considerable DOS piles up at two *E*_F_-symmetric gap edges of about ±19 meV. These characteristics, hallmarks of fully gapped superconductivity, suggest no gap node in the superconducting gap function of SNCO on the Fermi surface. At elevated temperatures, the superconducting gap is progressively smeared out and vanishes at 78 K (see the red curve). Note that, albeit weak (green curve), the gap survives above the observed *T*_c_ maximum of ∼30 K as shown in the electrical transport measurements in Fig. 1a, which we here ascribe to a spatial inhomogeneity of *T*_c_ inside the SNCO films.

**Figure 2.**
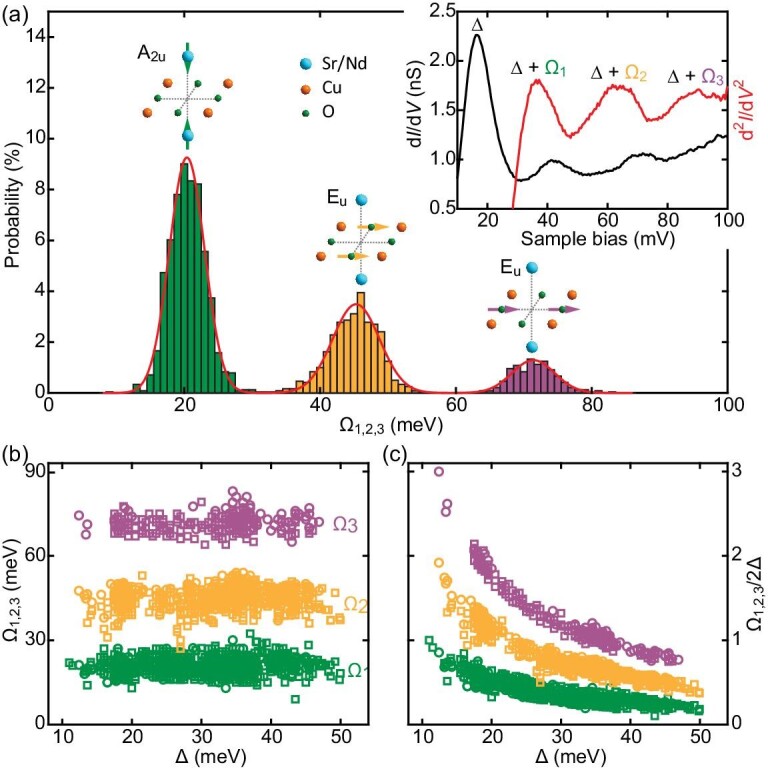
Lattice vibrational modes. (a) Histogram of measured bosonic mode energies Ω from a sequence of d*I*/d*V* spectra in nine similar SNCO samples with *x* ∼ 0.10. By fitting the data to a multipeak Gaussian function, three distinct bosonic modes at Ω_1_ = 20 ± 3 meV, Ω_2_ = 45 ± 4 meV and Ω_3_ = 72 ± 3 meV are obtained, with their energies close to those of the external (green arrows), bending (yellow arrows) and stretching (purple arrows) phonons of copper oxides [22,23]. Here the statistical errors of Ω_1,2,3_ indicate the full width at half maximum of the corresponding Gaussian peaks. Inserted in the top right corner are a representative superconducting spectrum (setpoint: *V* = −200 mV and *I* = 100 pA) and its derivative in the empty states, from which Ω_1,2,3_ can be readily extracted. (b) and (c) Variation of the bosonic mode energies Ω_1,2,3_ and Ω_1,2,3_/2Δ with the spatially varying gap magnitude Δ. Two different symbols of circles and squares denote data extracted from the occupied and empty states, respectively.

Careful measurements on various samples and superconducting regions indicate that the nodeless electron pairing occurs universally on the CuO_2_ planes of SNCO, irrespective of the spatial inhomogeneity in Δ (Figs S1 and S2 in the online supplementary material). This finding turns out to be consistent with previous tunneling and angle-resolved photoemission studies of a sister compound Sr_0.9_La_0.1_CuO_2_ [19,20]. Furthermore, multiple peak-dip-hump fine structures develop frequently (>75%) outside the superconducting gaps, which are pairwise centered at *E*_F_ and smear out at elevated temperatures (Fig. 1d). These traits were commonly interpreted as the signatures of bosonic excitations in superconductors [4,10–12]. Tunneling spectra on the bosonic mode energy Ω and its correlation with Δ can potentially distinguish between candidates for the pairing glue [10–12,21]. Inserted in Fig. 2a are one representative superconducting spectrum (black curve) and its derivative (d^2^*I*/d*V*^2^, red curve), only showing the empty states. By taking the maxima in the second derivative of conductance d^2^*I*/d*V*^2^ as estimates of the energies *E* = Δ + Ω, three bosonic modes with energies at Ω_1,2,3_ are extracted. A statistical estimate of Ω from all measured superconducting d*I*/d*V* spectra in both empty and occupied states yields thousands of independent observables, whose histograms are plotted in Fig. 2a. The average mode energies are measured to be Ω_1_ = 20 ± 3 meV, Ω_2 _= 45 ± 4 meV and Ω_3 _= 72 ± 3 meV, respectively. Evidently, Ω_2_ and Ω_3_ are not multiples of Ω_1_ (Fig. S3 in the online supplementary material), which excludes the possibility that they are caused by a harmonic multi-boson excitation of the same mode Ω_1_. This appears to be in good agreement with the intensity differences of Ω_1,2,3_ (Fig. 1d, and Fig. S1 in the online supplementary material). For some spectra, the bosonic modes Ω_2_ and Ω_3_ are too faint to be easily read out, leading to their probabilities being relatively lower than Ω_1_ in the histograms of Ω_1,2,3_ (Fig. 2a).

The correlations between the spatially resolved Ω_1,2,3_ and Δ are plotted in Fig. 2b and c. Despite a substantial spatial variation in Δ (Fig. S2 in the online supplementary material), the bosonic mode energies Ω_1,2,3_ alter little and the local ratio of Ω_1,2,3_ to 2Δ (Ω_1,2,3_/2Δ) exceeds unity for small Δ. Both findings run counter to the scenario of spin excitations, whose energies are generally dependent on Δ [11] and remain below the pair-breaking energy, to wit, Ω/2Δ < 1 [21]. By contrast, since energies of lattice vibrations change little with the doping level (i.e. Δ), they are natural candidates for the three bosonic excitations observed. Actually, the Ω_1,2,3_ show incredible coincidences with the external (∼20 meV), bending (∼45 meV) and stretching (∼72 meV) phonon mode energies, which were measured independently by optics [22] and Raman [23] in bulk SrCuO_2_. It should be stressed that this concurrent observation of the three key phonon modes from one spectrum is rather challenging in cuprate superconductors. We attribute this unprecedented success to a rare new measure of the major CuO_2_ planes.

### Nanoscale electronic phase separation

**Figure 3.**
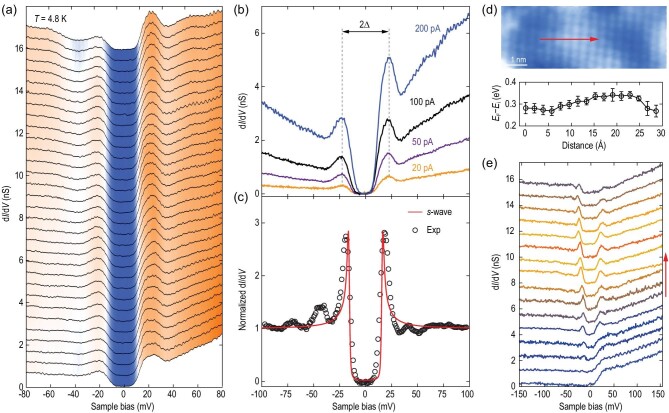
Tunneling spectra and nodeless superconductivity on CuO_2_. (a) Line-cut d*I*/d*V* spectra taken at equal separations (0.05 nm) in one superconducting domain. Setpoint: *V* = −100 mV and *I* = 100 pA. (b) Tip-to-sample distance dependence of d*I*/d*V* spectra at a specific position. The tunneling current *I* is changed from 20 pA (large distance, bottom curve) to 200 pA (small distance, top curve) at a constantly stabilized *V* = −100 mV. (c) Normalized d*I*/d*V* spectrum with pronounced coherence peaks at 4.8 K (circles) and its best fit (red curve) to a single *s*-wave superconducting gap with Δ = 17 meV. The normalization was performed by dividing the raw d*I*/d*V* spectrum by its background, which was extracted from a quadratic fit to the conductance for |*V*| > 50 mV. (d) An 8 nm × 3 nm STM topography (*V* = −1.0 V, *I* = 20 pA) showing nanoscale phase separation between superconducting (bright) and insulating (dark) domains. The bottom panel shows *E*_F_ shifts relative to *E*_i_ along the red arrow. Here a larger *E*_F_ − *E*_i_ means heavier electron doping. (e) A series of d*I*/d*V* spectra acquired along the red arrow in (d), plotted from bottom to top. Setpoint: *V *= 200 mV and *I* = 100 pA.

To eliminate any possible artifacts in our measurements, we collected spatially resolved tunneling d*I*/d*V* spectra over many regions of the samples and checked the tunneling junction quality. A representative set of d*I*/d*V* spectra in the superconducting region exhibit superior robustness of the nodeless pairing, coherence peaks and peak-dip-hump line shapes (Fig. 3a). Figure 3b compares a series of site-specific d*I*/d*V* spectra taken as a function of increasing the tip-to-sample distance (from top to bottom). The full superconducting gap remains essentially unchanged at any tunneling current, as anticipated for ideal vacuum tunneling. It was also found that a fraction of superconducting gaps display pronounced coherence peaks and could be fitted by the Dynes model using a single *s*-wave gap function [24], as exemplified in Fig. 3c. A minor discrepancy occurs between measured (black circles) and fitted (red curve) curves in the superconducting gap and suggests excess subgap DOS, whose origin merits further study. Figure 3d exhibits another region in which a set of spatially dependent d*I*/d*V* spectra (Fig. 3e) were taken along the red arrow. Note that the atomically resolved STM topography of Fig. 3d was acquired at −1.0 V, just around the charge-transfer band (CTB) onset of CuO_2_ in the electron-doped SNCO films [16,17]. The STM contrast mainly has an electronic origin (Fig. S4 in the online supplementary material). Domains mapped as bright correspond to the regions with relatively heavier neodymium dopants and thereby more emergent in-gap states (IGSs), and vice versa [16,25]. A local measurement of *E*_F_ relative to the midgap energy *E*_i_ (i.e. the center of charge transfer gap) of CuO_2_ [16], by exploring the spatial dependence of wider-energy-ranged d*I*/d*V* spectra in Fig. S5, convincingly supports this claim. As verified in the bottom panel of Fig. 3d, the *E*_F_ – *E*_i_ value, a good indicator of the electron doping level of neodymium, appears to be larger in the bright regions than in the dark ones. Meanwhile, a crossover from the full superconducting gaps to gapless or somewhat insulating tunneling spectra is apparent as the STM tip moves from the bright regions to the dark ones (Fig. 3e). Such direct imaging of the nanoscale electronic phase separation, which proves the existence of a generic characteristic of the SNCO epitaxial films (Fig. S2 in the online supplementary material) and has been extensively documented in other copper oxide superconductors [26,27], offers a straightforward account for the unusual electrical resistivity behavior in Fig. 1a and b.

**Figure 4.**
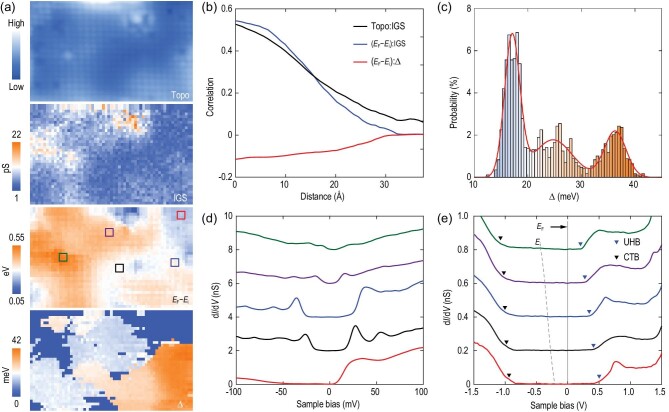
Spectroscopic mapping of nanoscale electronic phase separation. (a) Topography (10.5 nm × 6.2 nm, *V* = −0.8 V, *I* = 20 pA), spatial maps of IGSs, *E*_F_ – *E*_i_ and Δ extracted from a grid (61 pixels × 36 pixels) of spectroscopic data over the same field of view. The STM tip is stabilized at *V* = −1.6 V, *I* = 100 pA and *V* = −200 mV, *I* = 100 pA to measure the d*I*/d*V* spectra in the wide and narrow voltage ranges, respectively. (b) Angle-averaged cross-correlations of IGSs with the STM topography (black) and *E*_F_ – *E*_i_ (blue), as well as cross-correlation between the Δ and *E*_F_ – *E*_i_ (red). (c) Histogram of the superconducting gap Δ. Three discrete peaks from the multipeak Gaussian fit (red) arise from various superconducting domains with different doping levels. (d) and (e) Spatially averaged d*I*/d*V* spectra on the square-marked regions in the second lowest panel of (a), color coded to match with each other, measured in both narrow and wide energy scale ranges, respectively. The dashed line tracks the evolution of *E*_i_, which evenly separates between the CTB (black triangles) and upper-Hubbard band (UHB, blue triangles) of the CuO_2_ plane, while the gray solid line denotes *E*_F_.

In order to cast more light on the superconductivity of CuO_2_ at the nanoscale, we further mapped d*I*/d*V* spectra in both wide (from −1.5 to 1.5 V) and narrow (from −200 mV to 200 mV) voltage ranges, from which the local doping level and Δ can be readily extracted and compared in the same field of view. Data from such maps are shown in Fig. 4a. Here the spatial IGSs are estimated by integrating the spectral weights within the charge-transfer gap of the CuO_2_ plane, while the Δ is defined as half the separation between the two *E*_F_-symmetric superconducting coherence peaks. A direct visual comparison between the corresponding maps in Fig. 4a, and their cross-correlation in Fig. 4b, reveals that the larger *E*_F_ − *E*_i_ is correlated with topographically bright regions of populated IGSs on a short-length scale of ∼3 nm. By contrast, the correlation between the *E*_F_ − *E*_i_ value and Δ is too small to draw any unbiased opinion. A careful inspection shows a strong spatial inhomogeneity of Δ (Fig. 4c) that relies non-monotonically on the *E*_F_ − *E*_i_ value (Fig. 4d and e). The superconducting gap emerges at a certain threshold of *E*_F_ − *E*_i_ (i.e. the electron doping level), increases and then decreases in magnitude as the local doping level is increased further. This describes a primary source of the weak correlation between them (Fig. 4b). Such a non-monotonic variation of Δ with the local doping level is derived from one sample by taking advantage of the dopant-induced nanoscale electronic inhomogeneity and differs from the nodal *d*-wave gap behavior previously reported on the charge reservoir planes [2,8], which declines linearly with the chemical doping. Instead, it bears a resemblance to the dome-shaped (*T*_c_ versus doping) superconducting phase diagram of both electron- and hole-doped cuprates [1,2,28,29]. This indicates that the observed nodeless gaps on the CuO_2_ plane are intimately linked to the superconducting properties (*T*_c_) of cuprates.

## CONCLUSION

We have arrived at our key findings of superconducting CuO_2_ planes in SNCO, namely the nodeless electron pairing and spectroscopic evidence for the lattice vibrational modes in the superconducting domains. Although the *E*_F_ − *E*_i_ and Δ vary significantly from domain to domain, the superconducting gaps are always fully opened on the Fermi surface (Figs 3 and 4d, and Figs S1 and S2 in the online supplementary material). A statistical average of Δ from >3400 d*I*/d*V* spectra over many superconducting domains of all nine SNCO samples we studied (Fig. S1 in the online supplementary material) yields a value of 27 ± 8 meV. This mean gap size and the maximum gap Δ_max_ ∼ 40 meV observed in Fig. 4c stand out as the highest of records in all electron-doped cuprate superconductors [19,20,28], and are comparable to those reported in the hole-doped counterparts [29]. Under this context, equivalently high-*T*_c_ superconductivity might be potentially realized in the electron-doped infinite-layer cuprates once the inherent sample inhomogeneity is best minimized [30].

Our direct observation of nodeless superconductivity in the electron-doped cuprates of SNCO differs from the prior STM probe of a nodal *d*-wave gap function on the charge reservoir planes of various hole-doped cuprates [8]. Although it is tempting to examine the effect of an antiferromagnetic order on the nodeless energy gaps [20,31], our results exhibit much better consistency with those on the superconducting CuO_2_ planes [9,32,33]. It thus becomes highly desirable to revisit the role of charge reservoir layers during tunneling measurements of cuprates, and to find out whether the nodeless electron pairing is generic to the CuO_2_ planes of the copper oxide superconductors. Combined with the simultaneous measurements of lattice vibrational modes, which are indiscernible from tunneling spectra of the non-superconducting domains due to the vanishing paired electrons there (Fig. 4d), our results agree with a phonon-mediated *s*-wave pairing state in SNCO. However, caution is taken when explaining the findings using the conventional wisdom of Bardeen-Cooper-Schrieffer (BCS) theory. This is because of (i) the gap-to-*T*_c_ ratio 2Δ/*k*_B_*T*_c_ ∼ 14 (Fig. 1d) that largely exceeds the weak-coupling BCS value of 3.53 and (ii) the anomalous dome-shaped doping dependence of Δ (BCS theory predicts no obvious dependence of Δ on doping). These unconventional features, which have been observed in fulleride superconductors [34] and monolayer FeSe films grown on SrTiO_3_(001) substrates [35–37], go beyond the weak-coupling BCS picture. They do not, however, necessarily violate a phonon-mediated superconducting state with the local non-retarded pairs [38]. From this point of view, our results demonstrate the vital significance of electron-lattice interaction in the superconductivity of infinite-layer cuprates [39]. A further measurement of the oxygen isotope effects on Δ and Ω helps understand the role of phonons in the observed nodeless superconductivity.

## MATERIALS AND METHODS

### Sample growth

High-quality SNCO (0.008 < *x* < 0.110) thin films were epitaxially grown in an ozone-assisted molecular beam epitaxy (O-MBE) chamber that contained a quartz crystal microbalance (QCM, Inficon SQM160H) for precise flux calibration. Atomically flat SrTiO_3_(001) substrates with different Nb doping levels of 0.05 wt% and 0.5 wt% were heated to 1200°C under ultra-high vacuum (UHV) conditions for 20 minutes to acquire a TiO_2_ terminated surface. The epitaxial SNCO films for STM and transport measurements were prepared on the 0.5 wt% and 0.05 wt% Nb-doped SrTiO_3_ substrates, respectively. As oxidant, the distilled ozone flux was injected from a home-built ozone system into the O-MBE chamber by a nozzle, ∼40 mm away from the substrates. All samples were grown by co-evaporating high-purity metal sources (Nd, Sr and Cu) from standard Knudsen cells under an ozone beam flux of ∼1.1 × 10^–5 ^Torr and at an optimized substrate temperature *T*_sub_ of 550°C [17]. The lower *T*_sub_ (<500°C) was revealed to degrade the sample quality of SNCO severely, while the higher ones (>610°C) resulted in another competing orthorhombic phase. After growth, the films were annealed in UHV at the identical *T*_sub_ for 0.5 hours and then cooled down to room temperature.

Prior to every film growth, we calibrated the beam flux of metal sources in sequence, to ensure the stoichiometry of SNCO films. The growth rate was kept at ∼0.4 unit cells per minute. The doping level *x* was nominally deduced by *in-situ* QCM by calculating the flux ratio between the Nd and Cu sources, with an experimental uncertainty of ∼0.5%. At the same time, the satellite peaks (Kiessig fringes) in the X-ray diffraction (XRD) spectra allowed us to estimate the film thickness [16,17], which agreed nicely with the nominal one deduced by the QCM-measured flux of Cu and growth duration.

### *In-situ* STM measurements

All STM measurements were performed in a Unisoku USM 1300S ^3^He system, which was connected to the O-MBE chamber, at a constant temperature of 4.8 K, unless otherwise specified. The system pressure was lower than 1.0 × 10^–10^ Torr. Polycrystalline PtIr tips were cleaned via *e*-beam bombardment and calibrated on MBE-prepared Ag/Si(111) prior to the STM measurements. The STM topographies were acquired in a constant current mode with the voltage applied on the sample. The differential conductance d*I*/d*V* spectra and maps were measured by using a standard lock-in technique with a small bias modulation at 937 Hz. The system grounding and shielding were optimized to increase the stability and spectroscopic energy resolution (∼1.0 meV) of our STM apparatus.

### Transport measurements

After *in-situ* STM characterization and *ex-situ* XRD measurements, the transport measurements were carried out in a standard physical property measurement system (PPMS, Quantum Design). Freshly cut indium dots were cold pressed onto the samples as contacts. The resistivity was measured in a four-terminal configuration by a standard lock-in technique with a typical excitation current of 1 μA at 13 Hz.

## Supplementary Material

nwab225_Supplemental_File
