# Supplementary material for: Direct observation of nodeless superconductivity and phonon modes in electron-doped copper oxide Sr1−xNdxCuO2
Source: Natl Sci Rev. 2021 Dec 15;9(4):nwab225. doi: 10.1093/nsr/nwab225 (PMC9070465; doi:10.1093/nsr/nwab225)
Supplement: nwab225_Supplemental_File [file nwab225_supplemental_file.pdf]

Supplementary Material for

Direct observation of nodeless superconductivity and phonon modes in electron-doped copper oxide $Sr_{1-x}Nd_xCuO_2$

Jia-Qi Fan[1], Xue-Qing Yu[1], Fang-Jun Cheng[1], Heng Wang[1], Ruifeng Wang[1], Xiaobing Ma[1], Xiao-Peng Hu[1], Ding Zhang[1,2,3,4], Xu-Cun Ma[1,2 †], Qi-Kun Xue[1,2,3,5 †], Can-Li Song[1,2 †]

[1]*State Key Laboratory of Low-Dimensional Quantum Physics, Department of Physics, Tsinghua University, Beijing 100084, China*

[2]*Frontier Science Center for Quantum Information, Beijing 100084, China*

[3]*Beijing Academy of Quantum Information Sciences, Beijing 100193, China*

[4]*RIKEN Center for Emergent Matter Science (CEMS), Wako, Saitama 351-0198, Japan*

[5]*Southern University of Science and Technology, Shenzhen 518055, China*

[†]*To whom correspondence should be addressed. Email: clsong07@mail.tsinghua.edu.cn, xucunma@mail.tsinghua.edu.cn, qkxue@mail.tsinghua.edu.cn*

This supplement includes:

Fig. S1 to S5

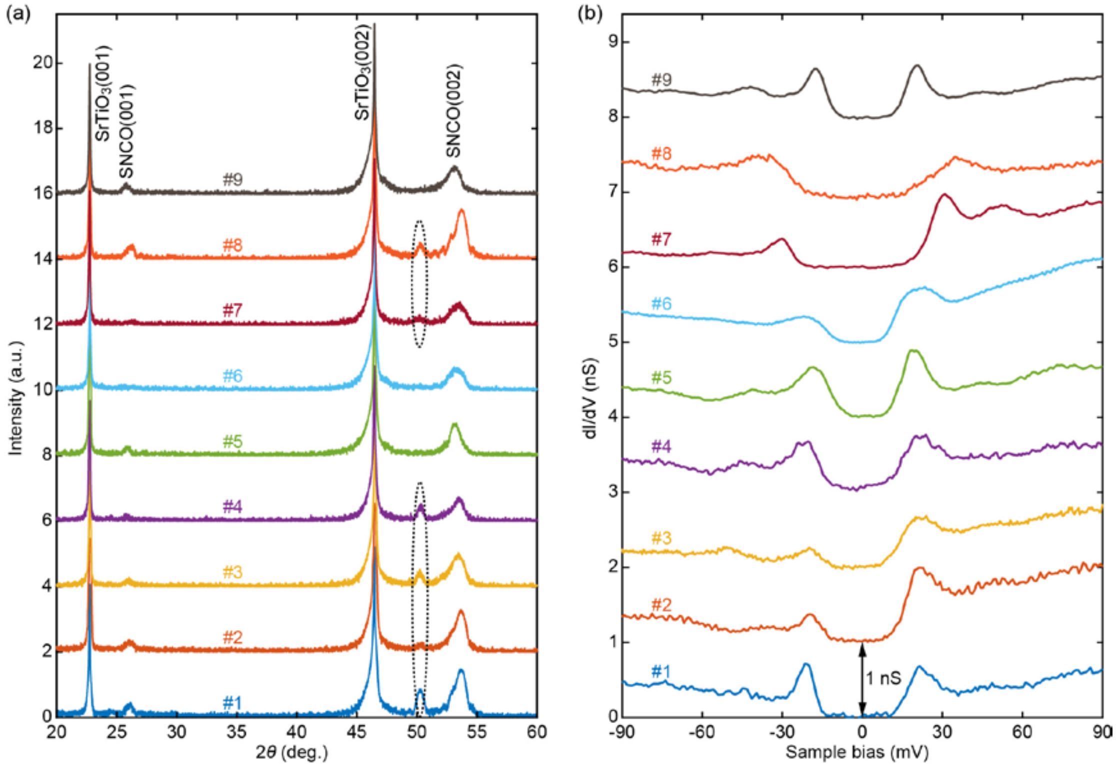

Figure. S1. Data reproducibility and robust nodeless superconductivity. (a) XRD spectra of nine similar SNCO samples (from #1 to #9) with a nominal doping $x \sim 0.100$ measured by using the monochromatic Cu $K_{\alpha 1}$ radiation with a wavelength of λ = 0.15406 nm. The experimental error of actual Nd dopant concentration results into a tiny trace of hole-doped SNCO (marked by the dashed ovals), caused by the appreciable intake of apical oxygens [16,17]. In this study, only the electron-doped SNCO regions have been explored with interest. (b) Tunneling dI/dV spectra consistently showing full superconducting gaps in various SNCO samples, color-coded to match the XRD spectra for the same sample in (a). Every curve corresponds to spatially averaged dI/dV spectrum in one typically superconducting domain. For clarity, the curves are vertically offset by 1 nS. The tunneling junction was stabilized at I = 100 pA and V = -200 mV, except for #1 (V = 200 mV), #4 (V = -150 mV), #5 (V = -250 mV) and #6 (V = -100 mV).

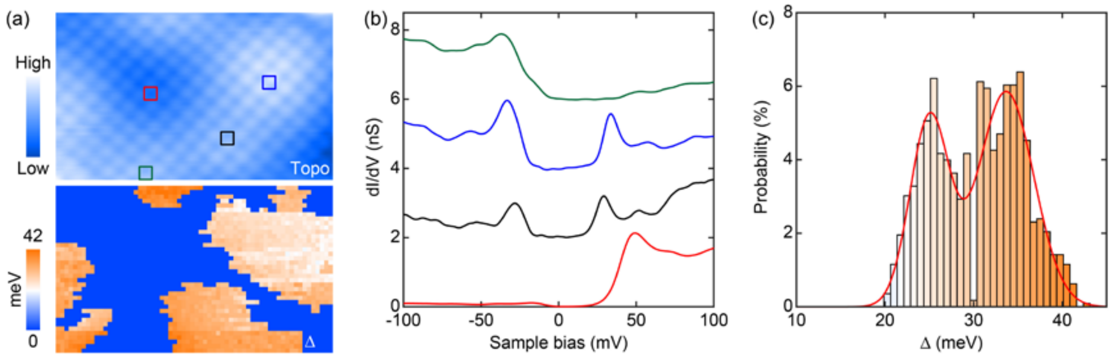

Figure. S2. Spectroscopic mapping of electronic phase separation and spatial inhomogeneity in Δ. (a) STM topography (7.0 nm × 4.2 nm, *V* = -0.8 V, *I* = 20 pA) and Δ map extracted from a grid (64 pixels × 38 pixels) spectroscopic data over the same field of view. The blue regions exhibit no spectroscopic sign of superconductivity, for which we assign Δ as zero. (b) Spatially-averaged tunneling dI/dV spectra on the square-marked regions in (a), color coded to match with each other. Setpoint: *V* = -200 mV and *I* = 100 pA. (c) Histogram of the measured superconducting gaps. Two discrete peaks of Δ from the multipeak Gaussian fit (red line) arise from different superconducting domains with varying doping levels.

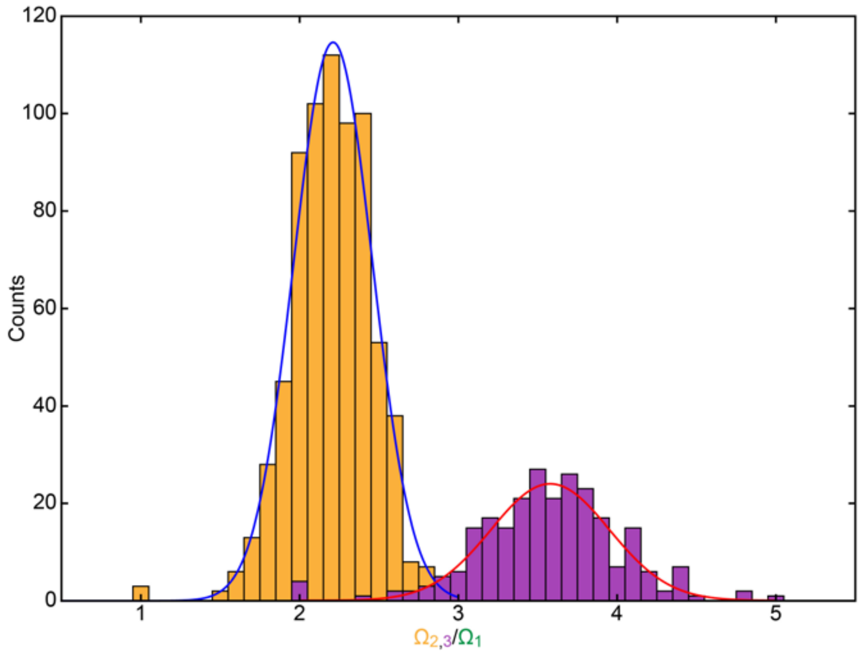

Figure. S3. Histograms of the mode energy ratio of Ω_2/Ω_1 (orange) and Ω_3/Ω_1 (purple). Each data has been carefully extracted from one identical spectrum with discernible Ω_2/Ω_1 and/or Ω_3/Ω_1. Blue and red solid lines denote Gaussian fits used to determine the ratio $\Omega_2/\Omega_1 = 2.21 \pm 0.01$ and $\Omega_3/\Omega_1 = 3.58 \pm 0.02$, respectively. The large deviations of Ω_2/Ω_1 and Ω_3/Ω_1 from integers exclude identifications of Ω_2 and Ω_3 as multiples of the same mode Ω_1.

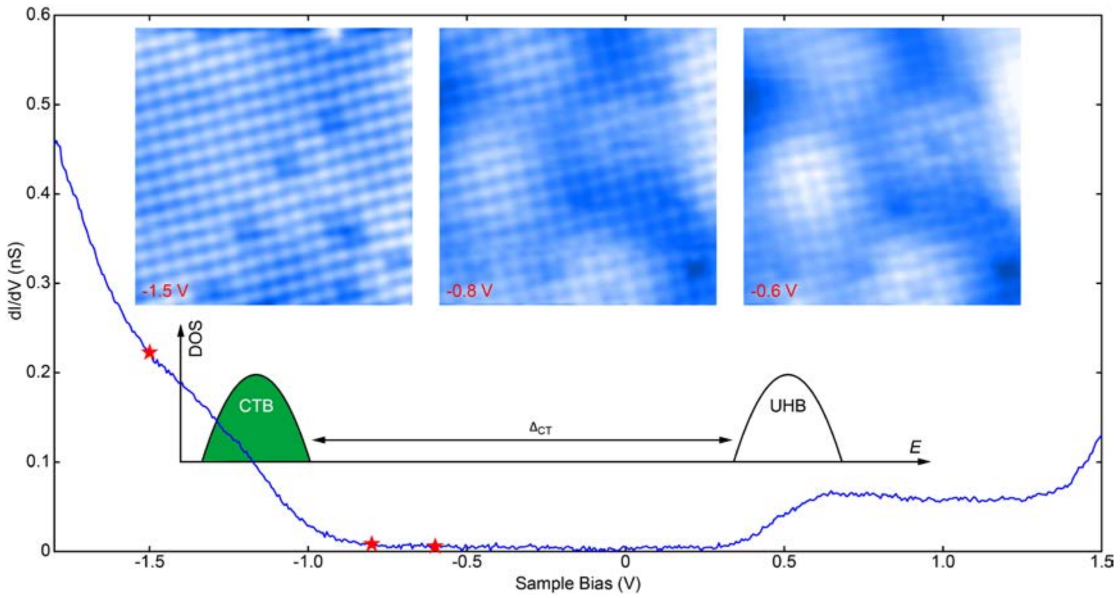

Figure. S4. Bias-dependent STM imaging contrast of CuO_2. Spatially-averaged dI/dV (blue curve) spectrum in one SNCO epitaxial film of $x \sim 0.100$, stabilized at V = -1.8 V, I = 100 pA. Inserted are bias-dependent STM topographies (6.2 nm × 6.2 nm, I = 20 pA) in the same field of view and the schematic band structure of pristine cuprate, only showing the UHB (unfilled) and CTB (green). The three red stars from left to right mark the sample biases of V = -1.5 eV, -0.8 eV and -0.6 eV applied to acquire the atomically-resolved STM topographic images in the top panel. Apparently, a nanometer-scale STM contrast becomes dominant for sample biases located within the charge-transfer gap (Δ_{CT}), while the STM topography measured at -1.5 V exhibit no such contrast. This strongly hints at an electronic origin of the corresponding STM imaging contrast, primarily caused by a local doping variation of trivalent neodymium.

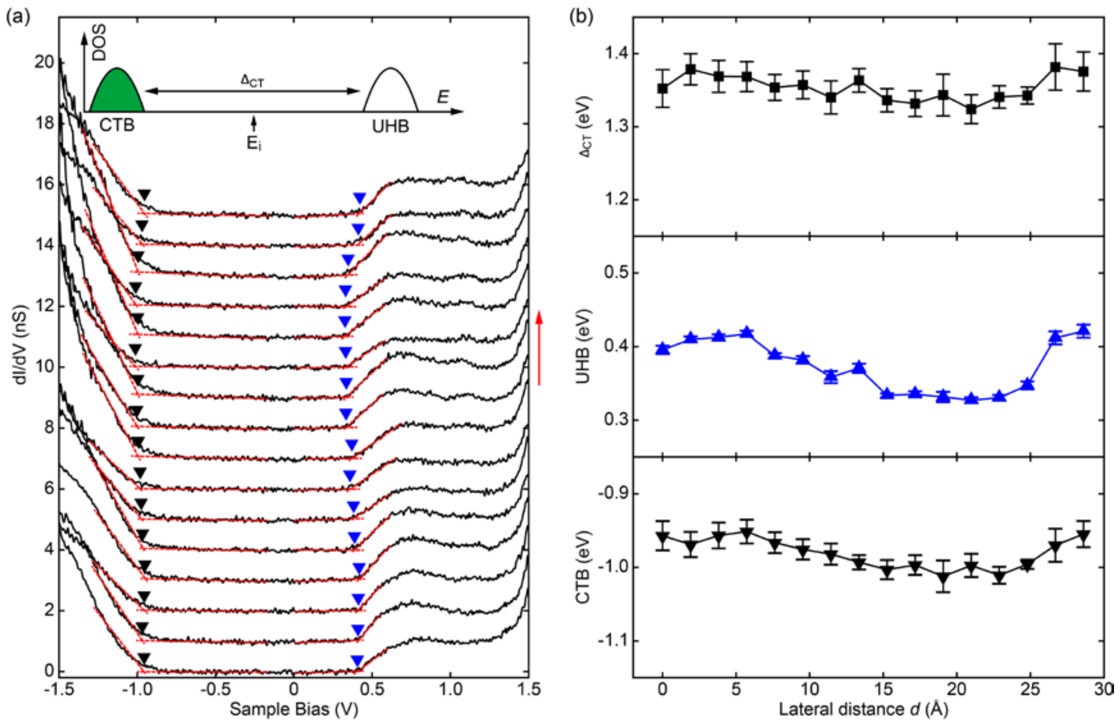

Figure. S5. Dopant-induced nanoscale electronic inhomogeneity and Mott parameters in SNCO. (a) Tunneling dI/dV spectra measured at equal separation (~ 0.18 nm) along the red arrow in Fig. 3(d), illustrating the spatial variations of the CTB (black triangles) and UHB (blue triangles) onsets on the CuO_2 plane of SNCO. The spectra were taken by stabilizing the setpoint at V = 1.5 V and I = 100 pA. Red dashed lines indicate the linear fits to the electronic DOS justly below and above CTB/UHB, and the points of interaction are defined as the CTB/UHB onset energies. Inserted are schematic energy bands of cuprates, showing the CTB (green), UHB (unfilled) and the midgap energy E_i (i.e. the center of charge transfer gap, see the black arrow). (b) Determined charge transfer gap Δ_{CT} (top panel), onset energies of UHB (middle panel) and CTB (bottom panel) as a function of the measured position d from bottom to top along the red arrow in (a). The error bars arise from the uncertainties of linear fits to the electronic DOS near the band edges for calculating the UHB and CTB onsets.
